# Holm multiple correction for large-scale gene-shape association mapping

**DOI:** 10.1186/1471-2156-15-S1-S5

**Published:** 2014-06-20

**Authors:** Guifang Fu, Garrett Saunders, John Stevens

**Affiliations:** 1 Department of Mathematics & Statistics, Utah State University, 3900 Old Main, Logan, UT, USA

**Keywords:** Bonferroni, Holm, QTL mapping, LD, Multiple correction

## Abstract

**Background:**

Linkage Disequilibrium (LD) is a powerful approach for the identification and characterization of morphological shape, which usually involves multiple genetic markers. However, multiple testing corrections substantially reduce the power of the associated tests. In addition, the principle component analysis (PCA), used to quantify the shape variations into several principal phenotypes, further increases the number of tests. As a result, a powerful multiple testing correction for simultaneous large-scale gene-shape association tests is an essential part of determining statistical significance. Bonferroni adjustments and permutation tests are the most popular approaches to correcting for multiple tests within LD based Quantitative Trait Loci (QTL) models. However, permutations are extremely computationally expensive and may mislead in the presence of family structure. The Bonferroni correction, though simple and fast, is conservative and has low power for large-scale testing.

**Results:**

We propose a new multiple testing approach, constructed by combining an Intersection Union Test (IUT) with the Holm correction, which strongly controls the family-wise error rate (FWER) without any additional assumptions on the joint distribution of the test statistics or dependence structure of the markers. The power improvement for the Holm correction, as compared to the standard Bonferroni correction, is examined through a simulation study. A consistent and moderate increase in power is found under the majority of simulated circumstances, including various sample sizes, Heritabilities, and numbers of markers. The power gains are further demonstrated on real leaf shape data from a natural population of poplar, *Populus szechuanica var tietica*, where more significant QTL associated with morphological shape are detected than under the previously applied Bonferroni adjustment.

**Conclusion:**

The Holm correction is a valid and powerful method for assessing gene-shape association involving multiple markers, which not only controls the FWER in the strong sense but also improves statistical power.

## Background

Linkage Disequilibrium (LD)-based Quantitative Trait Loci (QTL) studies now involve large-scale numbers of genetic markers and play a significant role in identifying underlying genetic variants for complex quantitative traits such as morphological shape or human disease [1-5]. A major issue for LD based QTL mapping is in determining significance levels for the testing of multiple individual markers. Three reasons add to the complexity of this multiple testing correction. First, new genotyping techniques make it common to measure tens of thousands of markers. The more statistical tests that we perform for identifying significant gene-trait associations, the more likely we are to reject the null hypothesis when it is true. This problem is also called the inflation of the type I error [6,7]. Second, high dimensional shape traits, often quantified by multiple principal components, dramatically increase the number of multiple tests by as much as three or more times [5,8,9]. Third, independence of test statistics is not guaranteed because correlations between markers lead to highly complicated and unknown dependency structures.

The Bonferroni correction, as one of the most popular multiple correction approaches, is known to be conservative and have low power for large-scale tests [10]. Permutations, although the current gold standard for assessing significance levels in genetic mapping studies with multiple markers, is extremely time consuming due to its computational burden, and may not work well if the population has family structure [6,11-13]. Therefore, it is necessary to seek alternative approaches that can improve the power for largescale simultaneous individual marker tests while preserving control of the family-wise error rate (FWER) under nominal significance thresholds (e.g. *α *= 0.05) [14,15].

In this article, we propose a uniformly more powerful sequentially rejective multiple testing approach that strongly controls the FWER for the LD based shape mapping model, by merging Holm's procedure [16] with the Intersection Union Test (IUT) [17]. The new procedure makes no assumptions on the joint distribution of the test statistics. The advantage of the Holm correction over the standard Bonferroni correction has been known to statisticians for over 35 years [16,18-20] but has not yet gained traction in LD based QTL mapping.

A critical challenge in large-scale LD association tests is the increase in the false positive rate if selected markers are not in complete LD with each other. In this case, the power is likely reduced (or false negative rate inflates) if the correction for multiple comparisons is overly conservative or if independence is assumed for markers with strong LD associations with each other. Despite the fact that the false discovery rate (FDR) is very popular and has been extensively used in multiple hypothesis testing [21], the FWER, the probability of making at least one type I error, exerts a more stringent control over the FDR. Since FDR is controlled only for all selected markers and provides no promise of control for an arbitrarily selected subset of the significant markers, researchers may detect more spurious QTLs using the FDR in place of the FWER as they often consider only a subset of the significant results [22]. Therefore, we recommend controlling the FWER rather than FDR whenever only the most promising results are valued, such as in LD based QTL mapping.

Detecting a significant shape QTL requires two hypothesis tests [5], the first testing for the association between QTL and shape, and the second testing for the LD between the observable marker and underlying QTL. Currently, Bonferroni corrections are applied separately to two families of hypotheses, one family consisting of the first hypothesis test for all markers, and the other family consisting of the second hypothesis test for all markers. Only those markers showing significance within both families, after correcting for multiple tests, are identified as linked to a QTL. This amounts to performing an IUT with the two test statistics for each marker and applying a Bonferroni correction for multiple markers. Although the LD based QTL model has been successful in locating significant QTLs [5,23,24], two improvements can be made within the multiple hypothesis testing aspect. First, several gene-shape association tests were made separately for each principal component (PC). Since these PCs quantify the original high dimensional shape variations from different directions, the multiple testing correction should account for these separate PCs as well as for multiple markers. Currently, multiple PCs are not accounted for in the multiplicity correction. Second, we introduce the uniformly more powerful Holm adjustment on the *p*-values resulting from the IUT, which shows greater power than the Bonferroni approach.

The significance of the power advantage of the Holm method over the Bonferroni method is demonstrated through both simulations and a real leaf shape data of a natural population of poplar, *Populus szechuanica var tietica*. We detect more significant QTL than were previously detected in the literature while still ensuring strong control of the FWER. Since sample size, Heritability, and number of markers all determine the power, we illustrate the power differences for Heritabilities of 0.1 and 0.4, sample size small (100), medium (300), and large (500), and number of markers changing from 1, 10, 50, 100, 500 to 1,000.

## Results

### Power simulation

We investigated a simulation study to quantify the power advantage of the Holm adjustment over the standard Bonferroni adjustment within the LD based QTL mapping model of [5]. The QTL, phenotype, and markers were generated under the assumptions of the alternative hypotheses in (3) and (4). The QTL was generated using an assigned probability of *q *= 0.7 for the major allele. For each individual *i, Qi *= *l *with *l ∈ {*1, 2, 3*} *was used to code the QTL genotypes of *aa, Aa*, and *AA*, respectively. The normally distributed phenotype dependent on the value of the QTL is generated as *Y_i_|*(*Q_i _*= *l*) *~ N *(*µl, σ*). The means for the phenotype *Y *corresponding to the values of the QTL were set at *µ*_1 _= 8, *µ*_2 _= 10 and *µ*_3 _= 12. markers were then generated using the conditional probability of the marker genotype given the value of the QTL genotype for each individual. In general, for an LD based QTL mapping model, researchers genotype the marker first and then use the marker to generate a QTL based on the conditional probability of QTL genotype given marker genotype. However, for our purposes, we are interested in extending from single marker mapping to multiple marker mapping. Therefore, we derive the conditional probability of marker genotype given QTL genotype (see Table 1) from the Bayes Rule in Equation (1) and Table 2.

**Table 1:** The theoretical conditional probabilities of marker genotype (columns) given QTL genotype (rows).

	*MM*	*Mm*	*mm*
*AA*	*$\frac{p_{11}^{2}}{q^{2}}$*	*$\frac{2p_{11}p_{01}}{q^{2}}$*	*$\frac{p_{01}^{2}}{q^{2}}$*
*Aa*	*$\frac{2p_{11}p_{10}}{q\left(1-q\right)}$*	*$\frac{2\left(p_{11}p_{00+}p_{10}p_{01}\right)}{{\left(1-q\right)}^{2}}$*	*$\frac{2p_{00}^{2}}{{\left(1-q\right)}^{2}}$*
*aa*	*$\frac{p_{10}^{2}}{{\left(1-q\right)}^{2}}$*	*$\frac{2p_{10}p_{00}}{{\left(1-q\right)}^{2}}$*	*$\frac{p_{00}^{2}}{{\left(1-q\right)}^{2}}$*

**Table 2:** The theoretical joint distribution probabilities of marker and QTL haplotypes.

	AA	Aa	aa
MM	$p_{11}^{2}$	$2p_{11}p_{10}$	$p_{10}^{2}$
Mm	$2p_{11}p_{01}$	$2\left(p_{11}p_{00}+p_{10}p_{01}\right)$	$2p_{10}p_{00}$
mm	$p_{01}^{2}$	$2p_{01}p_{00}$	$p_{00}^{2}$

a

(1) $$P\left(\text{M|QTL}\right)=\frac{P\left(\text{QTL}\right)}{P\left(\text{QTL}\right)}.$$

Sample sizes of *n *= 100, 300, and 500 were used to represent small, medium, and large sample sizes, respectively. The number of markers per simulation was set at *m *= 1, 10, 50, 100, 500, and 1,000 to show the initial power under the single marker scenario and the corresponding decreasing power trend as the number of markers increases. Finally, the heritability was set at two values, *H*^2 ^= 0.1 and 0.4, corresponding to high and low error variance [25]. The model error variance σ^2 ^was computed using the heritability and genetic variance of the QTL. Power estimates were averaged over 1,000 simulations.

**Figure 1:**
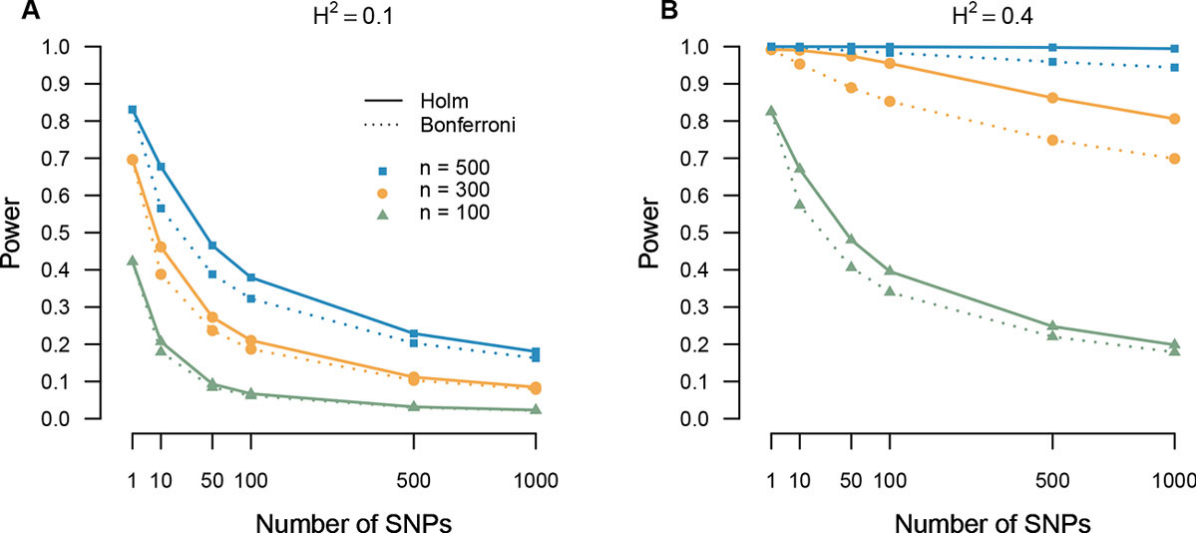
**Power comparison between the Holm adjustment and standard Bonferroni adjustment under different sample size, number of markers, and heritability (**A**: *H*^2 ^= 0.1, **B**: *H*^2 ^= 0.4)**.

The simulation results, depicted in Figure 1 and shown in Additional file 1, demonstrate the power comparison of the Holm adjustment with the traditional Bonferroni adjustment. These results provide an experimental reference for researchers about how power varies among different sample size *n*, the number of markers *m*, and the degree of heritability (*H*^2^). As expected, the power under high heritability (B: *H*^2 ^= 0.4) is much higher than that of the low heritability (A: *H*^2 ^= 0.1) and the power under large sample size (*n *= 500, blue curves) is much higher than that of the small sample size (*n *= 100, green curves). Under high heritability (*H*^2 ^= 0.4) and a larger sample size (*n *= 500), the power of the Holm multiplicity adjustment remains high, at least 99%, as the number of markers vary from 1 to 1,000. However, in practice it is often expensive to collect so many sample measurements, so these results are useful in deciding the opportunity costs in power for smaller sample sizes. It is worth noting that for moderate numbers of markers, the power increase of the Holm over the Bonferroni adjustment allows for maintaining the same power level of the Bonferroni adjustment while decreasing the sample size of the study or increasing the number of markers, a great advantage for researchers. For example, with a medium sample size of 300, the Holm correction maintains a power of 95% for 100 markers. Even when number of markers increase to 1, 000, the power achieved by the Holm correction is still as high as 80%.

Although the power increase of the Holm adjustment improves moderately over the standard Bonferroni adjustment for the case of high heritability (*H*^2 ^= 0.4) when the sample size is small (*n *= 100), these findings are comparable to seminal results found by previous multiplicity improvements over their competitors [16,21]. For the worst case when the data has extremely large variance (*H*^2 ^= 0.4) and relatively much smaller sample size (*n *= 100), the improvement of Holm over Bonferroni is not obvious. However, it is not an issue of multiple hypothesis testing but an issue of the least sample size necessary to guarantee a decent level of power.

All in all, the Holm method generally shows a valuable increase in power over the Bonferroni adjustment under the majority of simulated circumstances, including different combinations of sample size, numbers of markers, and Heritability. As long as the sample size is reasonably large in comparison to the variance to guarantee decent power, the improvement of the Holm correction over the Bonferroni is consistently meaningful.

### Poplar leaf shape QTL mapping project

To show how the power advantage of the Holm approach leads to increased scientific discovery over the Bonferroni adjustment, we apply it to a real poplar leaf shape QTL mapping study [5]. The study design used a representative leaf from each of 106 poplar trees (i.e., *Populus szechuanica *var. *tibetica *belonging to the Tacamahaca section) that was randomly selected and photographed for shape QTL analysis. The trees were also genotyped for a panel of 29 microsatellite markers (16 of them were considered). A Radius Centroid Contour (RCC) approach was used to represent the leaf shape (phenotype) with a high dimensional curve. The first three principal components (PCs) were selected to capture the majority variation of leaf shape from different directions to quantify the original high dimensional shape curves respectively. Significant QTLs affecting the shape variability (i.e., affecting the most important PCs) were mapped through the statistical LD based QTL mapping model [5]. Previously, the standard Bonferroni adjustment was used to control the FWER for the multiple markers [5, Table 1]. However, the researchers did not consider the multiple testing correction issue introduced by multiple PCs and their reported results treated as a family of hypotheses only the multiple markers within each PC. After including the multiple PCs within the family of interest, we found slightly different results, even under the previously applied Bonferroni correction.

**Figure 2:**
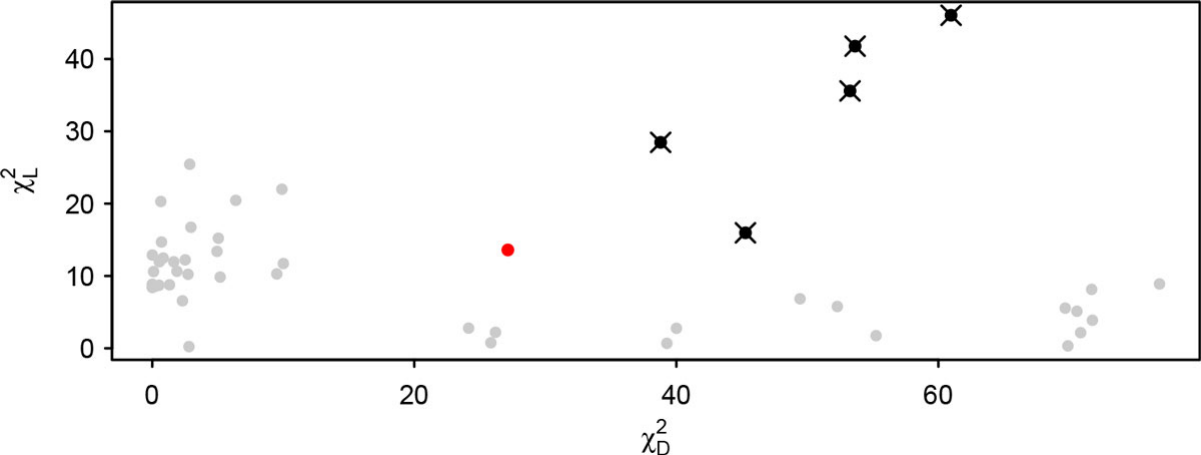
**Bivariate plot of the two test statistics $\chi_{L}^{2}$ and $\chi_{D}^{2}$**. Those points corresponding to markers identified as significant under the Holm correction are in black dots. Those identified significant by the standard Bonferroni correction are marked with an *×*. The red dot is the marker that is newly detected by the Holm correction. All other (non-significant) empirical joint test statistic points for multiple PCs and multiple markers are plotted in gray.

After applying our proposed Holm approach to provide a comprehensive multiple correction, not only including the multiple PCs in the correction but also including multiple markers within each PC, the Holm correction successfully detects all significant microsatellites that were detected by the Bonferroni correction. Further, the Holm correction detects one more marker, marker 10, that was not detected previously. Figure 2 demonstrates the bivariate plot of the two test statistics $\chi_{L}^{2}$and $\chi_{D}^{2}$. Those points corresponding to markers identified as significant under the Holm correction are in black dots. Those identified significant by the standard Bonferroni correction are marked with a ×. The red dot is the marker that is detected newly by Holm. All other (non-significant) empirical joint test statistic points for multiple PCs and multiple markers are plotted in gray. The new detected marker under the Holm correction is reasonable because it has similar test statistics value for the *H*0*L *: *µ*1 = *µ*2 = *µ*3 test but lower value for the *H*0*D *: *D *= 0 test, as compared to its nearest significant neighboring marker. It is well known that the critical threshold for the linkage tests are mostly somewhere around 10. Even if 1,000 multiple tests are considered, i.e., a significance level of 0.05*/*1000, the critical threshold of *χ*2 is at most 16.44. In this real shape data, the total number of tests that we performed is only 48 (16 markers and 3 PCs total) corresponding to the threshold of 10.752. Therefore, it is reasonable to call a marker significant with a test statistic value of 25 for *H*0*D *and a value for *H*0*L *is not lower than its nearest significant neighboring marker.

**Figure 3:**
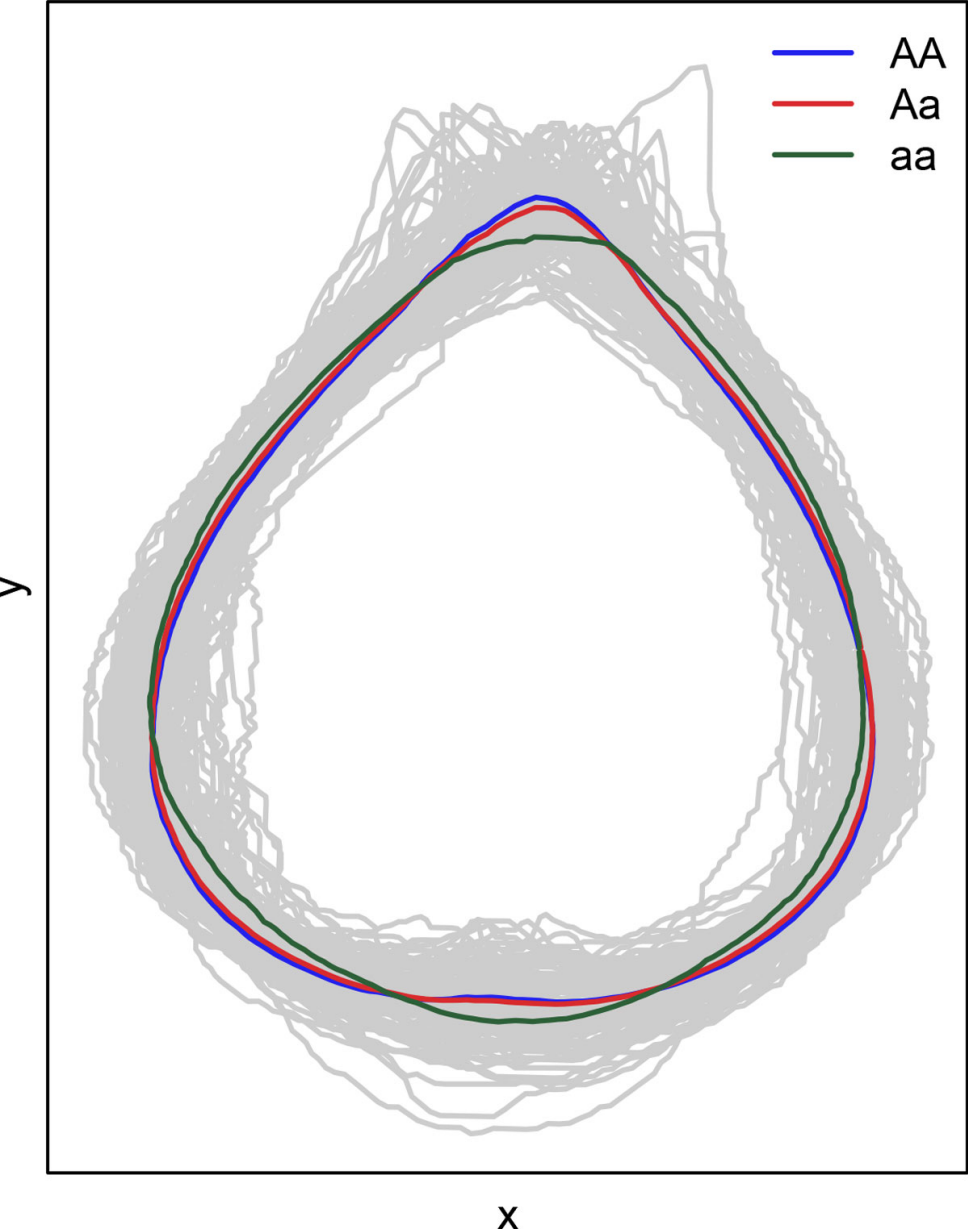
**The genotypic shape effects according to the different genotypes (AA, Aa, aa) of the QTL identified by marker 10 on PC 3**. This shows the increased sensitivity of the Holm approach as the effect of the aa QTL genotype is noticeably different from that of genotypes AA and Aa, but nevertheless, corresponds to information which was not detected under the Bonferroni correction.

**Figure 4:**
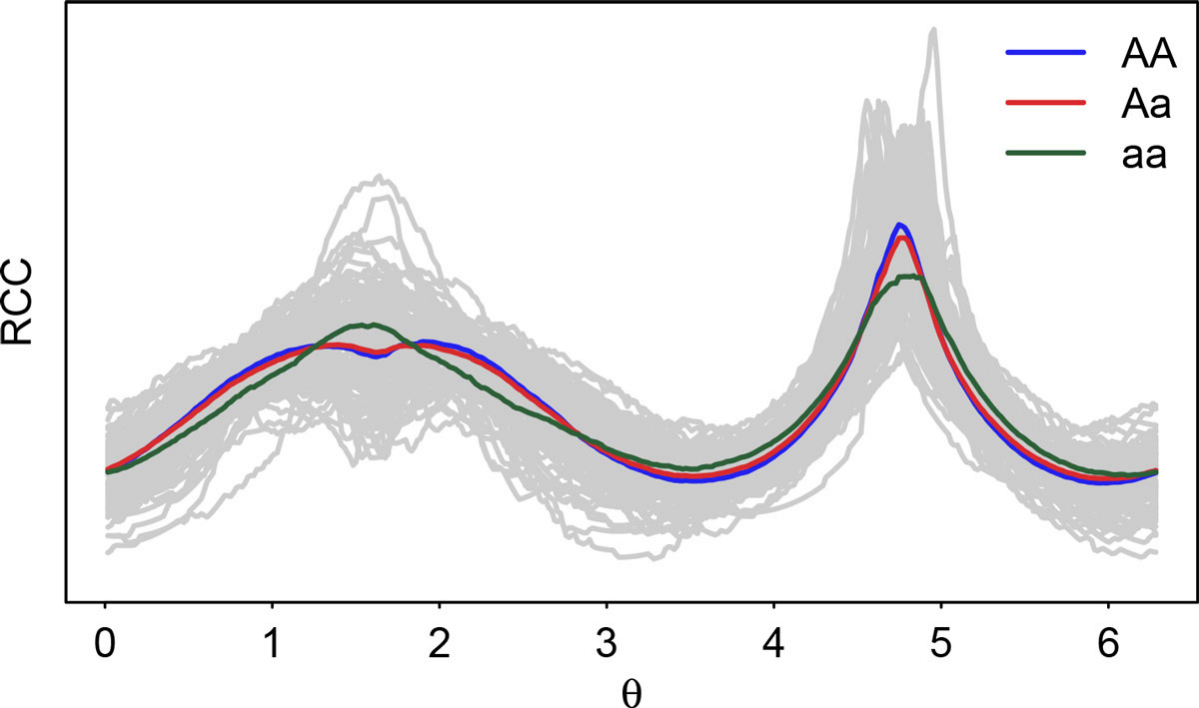
**RCC curves of leaf shape as a function of radial angle *θ *explained by the different genotypes (AA, Aa, aa) of the QTL identified by marker 10 on PC 3**. This shows the increased sensitivity of the Holm approach as the effect of the aa QTL genotype is noticeably different from that of genotypes AA and Aa, but nevertheless, corresponds to information which was not detected under the Bonferroni correction.

Figure 3 illustrates the genotypic shape effects according to the different genotypes (AA, Aa, aa) of the QTL identified by marker 10 on PC 3. It is evident that the effect of aa produces shorter leaf tips and more degrees of deltoidness at leaf base compare to the other genotypes. The effect of AA and Aa are very similar, which indicates a dominance effect. Although significant genotype differences can be observed visually, it is nevertheless not detected under the Bonferroni correction. This confirms the practical relevence of the increased sensitivity of the Holm correction over the Bonferroni. Figure 4 illustrates the RCC curves of leaf shape as a function of radial angle *θ *explained by the different genotypes (AA, Aa, aa) of the QTL identified by marker 10 on PC 3. It is evident that the RCC curve of aa has a higher peak when *θ *is close to *π/*2 but a lower dynamic pattern when *θ *is close to 3*π/*2, which matches the interpretations of above Figure 3 visualized from the leaf image domain. We believe that the advantages of our proposed approach will be more remarkable for a larger number of markers.

## Conclusion

Detecting significant genes that affect complex traits such as shape or disease through LD based QTL mapping has been popular in many disciplines [1-5,26-33]. The new genotyping techniques make it possible to simultaneously consider tens of thousands of markers, bringing substantial challenges for multiple testing. In addition, high dimensional shape traits, often involving multiple PC components, have been widely used and add yet another demand for a powerful and computationally efficient approach to adjust for multiple tests [5,8,9].

These multiple tests require an adjustment on the resulting *p*-values in order to preserve control of the family-wise error rate (FWER) at a pre-specified level *α*. Making the alpha level more stringent will create less errors, but it may lower the chance of detecting more real effects [7]. The FDR has been widely used as the error rate of interest. Typically however, a subset of the significant results are directly reported and therefore the FWER is the more desirable form of error rate to control [22]. The current standard approach in LD based QTL mapping is to apply a Bonferroni adjustment to correct for multiplicity and preserve control of the FWER. As is well known, the Bonferroni correction is overly conservative for large numbers of tests, but the advantages of simplicity without independence assumptions on the corresponding family of tests continue to make it popular. Permutation, although has been the gold standard for assessing significance levels in studies with multiple markers, is extremely time consuming, computationally intensive, and may not work well if the population has family structure [6,11-13].

In this article, we propose an uniformly more powerful multiple correction approach by integrating Holm [16] with the IUT test, which is assured strong control of the FWER under arbitrary dependencies among the test statistics. The advantage of Holm over Bonferroni actually has been recognized to statisticians for over 35 years [16,18-20] but is new to LD based QTL mapping. The significance of the power advantage of the Holm correction over the Bonferroni, has been established theoretically [16]. This work demonstrates the power advantage in LD based QTL mapping empirically through both simulation study and real data. As long as the sample size is reasonablly large in comparison to the variance to guarantee a decent power, the improvement of the Holm correction over the Bonferroni is consistent and meaningful.

## Methods

### LD based QTL model

To map the rough location of the QTL regulating shape, we apply the mixture model of [5]. Under this model, QTL mapping is accomplished by statistically modeling the genotypic variation through not only the association between phenotype and the putative QTL, but also the LD between the putative QTL and marker. Since the marker genotype is observable, the probabilities of a putative QTL genotype can be inferred by the conditional probability of QTL genotype (A) given the marker genotype (M), as long as there exists LD between the marker and putative QTL [5].

The mixture model of [5] assumes each individual's phenotype *Yi, i *= 1*, . . . , n*, is a random variate from density *f_l_*(*Y_i_|θ_l_*), where *l ∈ *{1, 2, 3} denote three distinct QTL genotypes. Each QTL genotype is assumed to induce a separate distribution of phenotypes. Typically, normal distributions are assumed for each *f_l_*(*Y_i_|θ_l_*) with *θ_l _*= (*µ_l_, σ*). From these assumptions, the corresponding likelihood is expressed as [5]

(2) $$L\left(\omega ,\mu ,\sigma \text{|}Y,M\right)=\overset{n}{\underset{i=1}{\prod }}\overset{3}{\underset{i=1}{\sum }}\omega_{l}{\text{|}}_{i}f_{l}\left(Y_{i}\text{|}\mu_{l},\sigma \right)$$

where *ωl|i *is the conditional probability of individual *I *having QTL genotype *l *given their marker genotypes, *µl *is the phenotypic mean for QTL genotype *l, σ *is the common variance for all genotypes, and *fl*(*Yi|µl, σ*) is the probability density of observations for individual *I *at QTL genotype *l *[5,25,34].

The probability of the marker's major allele (M) is denoted by *p*, and correspondingly 1 *− p *for the minor allele (m). Similarly, the probability of the QTL's major allele (A) is denoted by *q*, and correspondingly 1*−q *for the minor allele (a). Together, the marker and QTL form four haplotypes (MA, Ma, mA, and ma) with corresponding frequencies *p*_11 _= *pq*+*D, p*_10 _= *p*(1*−q*)*−D, p*_01 _= (1*−p*)*q −D*, and *p*_00 _= (1*−p*)(1*−q*)+*D*, respectively. Here, *D *is the linkage disequilibrium between marker and QTL. The conditional probabilities *ω_l|i _*of the QTL's various genotypes (AA, Aa, and aa) can be calculated upon the observed marker genotypes (MM, Mm, and mm) from the joint probabilities in Table 2[25,5]. The EM algorithm is then applied to the likelihood in (2) to obtain maximum likelihood estimates for all parameters [5,25].

### Two hypothesis tests

Through the likelihood in (2), the hypotheses

(3) $$H_{0}^{L}:\mu_{1}=\mu_{2}=\mu_{3}\;\text{vs}$$

$H_{1}^{L}:$ one of the equalities above does not hold can be used to test if the QTL is significantly associated with phenotype *Y *(i.e. existence of QTL). Since all the unknown parameters in (2) were estimated by maximum likelihood estimates (MLEs), a log likelihood ratio statistic can be used to test the hypotheses in (3) [5]. The resulting test statistic ($\chi_{L}^{2}$) is asymptotically distributed as a $\chi_{2}^{2}$ under $H_{0}^{L}$ for large enough samples.

On the other hand, linkage disequilibrium, denoted by *D*, between the marker and QTL can be tested by means of the hypotheses

(4) $$H_{0}^{D}:D=0\;\text{vs}\;H_{1}^{D}:D\neq 0.$$

The test statistic used to judge whether or not the QTL is significantly associated with marker is [5,35]:

(5) $$\chi_{D}^{2*}=\frac{n{\hat{D}}^{2}}{\hat{p}\left(1-\hat{p}\right)\hat{q}\left(1-\hat{q}\right)}$$

(6) $$=n{\hat{r}}^{2}.$$

Here, ${\hat{r}}^{2}$ is the square of the correlation coefficient between the marker and QTL that has been used in most of the related literature, which has many good sampling properties [36,37]. Under $H_{0}^{D}$, $\chi_{D}^{2}$ is asymptotically distributed as $\chi_{1}^{2}$, from which the tail probability (p-value) of the observed level of association can be determined [3,23,35,38].

In general, the Intersection-Union test (IUT) is defined as [39]

$$H0:\theta \in \cup_{\gamma \in \Gamma }\Theta_{\gamma }.$$

Here Γ is a finite or infinite set containing index of tests, *θ *is the unknown parameters under testing, and Θ*_γ _*specifies the statement of null hypothesis test for each index *γ*. Suppose that for each *γ ∈ *Γ, {*x *: *T_γ _*(*x*) *∈ R_γ_*} is the rejection region for each test $H_{0_{\gamma }}:\theta \in \Theta_{\gamma }$ versus $H1\gamma :\theta \in \Theta_{\gamma }^{c}$. Then the rejection region for the IUT test is

$$\cap_{\gamma \in \Gamma }\left\{x:T_{\gamma }\left(x\right)\in R_{\gamma }\right\}.$$

In the context of LD based QTL mapping, the tests of the above hypotheses (3) and (4) must be performed simultaneously to make the final conclusion, i.e., a significant QTL regulating shape is not detected unless both null hypotheses in (3) and (4) are rejected. $H_{0_{D}}$ : *D *= 0 is used to test the LD between QTL and marker, and $H_{0_{L}}:\mu_{1}=\mu_{2}=\mu_{3}$ is used to test the association between the phenotype and QTL, respectively. Thus, the IUT test with intersection rejection region but union null regions is appropriate for these two tests of each marker, resulting in a final set of *m *IUT *p*-values, where *m *is the number of markers tested. Then we integrate Holm into the IUT and perform multiplicity adjustment to these *m *IUT *p*-values, in place of the original Bonferroni correction that has been the current standard [5].

### The Holm correction

The Holm multiplicity correction [16] applies a "sequentially rejective Bonferroni test" to all currently non-rejected hypothesis in a step-down manner. The first step of our proposed approach is to use an IUT to obtain *m p*-values. Then, we order the tests from the one with the smallest *p*-value to the one with the largest *p*-value as *p*_(1)_*, . . . , p*_(*m*) _according to the usual order statistics notation. The smallest *p*-value, *p*_(1)_, corresponding to the ordered hypothesis *H*_(1)_, is then tested with the usual Bonferroni correction of *α*_1 _= *α/m*. If *H*_(1) _is declared significant, then the method continues by testing *p*_(2) _against *α*_2 _= *α/*(*m *− 1). So long as rejections continue to occur, *p*_(*i*) _is compared to *α_i _*= *α/*(*m − i *+ 1) until finally *p*_(*m*) _is compared to *α_m _*= *α*. If for any *i *∈ {1, 2*, . . . , m*} the hypothesis *H*_(*i*) _is not rejected, then the method stops and *H*_(*i*)_*, . . . , H*_(*m*) _are retained, i.e., not rejected. Therefore, the procedure stops when the first non-significant test is obtained or when all the tests have been performed.

To be exact, let *p*_(1)_*, . . . , p*_(*m*)_, denote the ordered *p*-values corresponding to the ordered *m *hypotheses obtained from IUTs, *H*_(1)_*, . . . , H*_(*m*)_. The ordered *p*-values for the test of *H*(*j*) are then compared to the thresholds *α_j _*where

(7) $$p_{\left(j\right)}\leq \alpha_{j}=\alpha /\left(m-j+1\right),$$

and all testings will stop at the *jth *test for which the first non-rejection occurs, i.e., the *j *for which *p*_(*j*) _*> α_j_*. Because the denominators are *m *− *j *+ 1 instead of *m*, Holm's procedure never rejects fewer hypotheses than the Bonferroni procedure does.

A multiple test procedure for testing hypotheses *H*_1_*, . . . , H_m _*is said to have *multiple level of significance α *(for free combinations) if for any non-empty index set *I ⊆ *{1, 2, 3*, . . . , m*} the supremum of the probability $P\left(\cup A_{i}^{c}\right)$ when *H_i _*are true for all *i ∈ I *is less than or equal to *α *where $A_{i}^{c}$ denotes the event of rejecting *H_i_*. This is called *strong control *of the FWER. A method that only controls the FWER under the assumption that all nulls are true has *weak control *of the FWER.

Importantly, as proved in [16], strong control of the FWER is ensured under the Holm adjustment, no matter the dependency structure of the corresponding test statistics. A simple, but elegant proof of the strong FWER control of this approach, under arbitrary dependence structures of the test statistics, is given in [16]. The idea behind the proof is as follows. Let *I *denote the set of indices corresponding to the true null hypotheses, and let *k *denote the number of hypotheses in *I*, so that *k ≤ m*. The well known Boole's Inequality shows that the FWER (the probability of at least one type I error) is controlled by the Bonferroni method so long as at most *α/k *is applied to the testing of all *k *true nulls. Specifically,

(8) $$\begin{array}{c} P\left(pi\leq \alpha \text{/}k,\text{for some }i\in I\right)\\ \;\;\leq \underset{i\in I}{\sum }P\left(pi\leq \alpha \text{/}k\right)\leq k\alpha \text{/}k=\alpha . \end{array}$$

Given the nature of the sequential testing of the Holm adjustment, at any stage *j *of testing, the number of true nulls remaining to be tested (*k*) will always be less than or equal to the number of hypotheses remaining to be tested (*m−j*+1). Hence, any true null will always be tested by at least *α/*(*m − j *+ 1) *≤ α/k*, ensuring strong control of the FWER no matter how many or which nulls are true.

Just as with the Bonferroni method, Holm's method is a distribution free approach to the multiple hypothesis testing issue. More importantly it is uniformly more powerful than the Bonferroni method as it will compare *P*_(2)_*, . . . , P*_(*m*) _to larger thresholds *α_i_, i *= 2*, . . . , m *than will the Bonferroni method. Therefore, it is clear that the Holm method should always be preferred over the Bonferroni method from a theoretical perspective. In the following sections, we will illustrate the benefit of Holm over Bonferroni from an application perspective.

## Competing interests

The authors declare that they have no competing interests.

## Authors' contributions

GF initiated the project, supervised the main ideas, closely guided several details, provided the estimation programming, wrote and revised the manuscript. GS participated in the development of the Holm method, made programs for multiple testing, performed data analysis, and drafted the manuscript. JS involved in idea development discussions, checked the validation of the method, and revised the manuscript.

## Supplementary Material

Additional file 1 Additional file 1 includes a single table showing the results of the power simulation as depicted in Figure 1. Click here for file
