# Supplementary material for: Holm multiple correction for large-scale gene-shape association mapping
Source: BMC Genet. 2014 Jun 20;15(Suppl 1):S5. doi: 10.1186/1471-2156-15-S1-S5 (PMC4118635; doi:10.1186/1471-2156-15-S1-S5)
Supplement: Additional file 1 — includes a single table showing the results of the power simulation as depicted in Figure 1. [file 1471-2156-15-S1-S5-S1.pdf]

Additional File 1

Table 1 The results of the power simulation as depicted in Figure 1.

	$n = 100$		$n = 300$		$n = 500$	
m	Bonferroni	Holm	Bonferroni	Holm	Bonferroni	Holm
$H^2 = 0.1$						
1	0.422	0.422	0.696	0.696	0.831	0.831
10	0.179	0.207	0.388	0.462	0.565	0.677
50	0.084	0.093	0.237	0.272	0.388	0.465
100	0.062	0.067	0.187	0.210	0.322	0.379
500	0.030	0.032	0.103	0.112	0.203	0.229
1000	0.022	0.023	0.079	0.085	0.163	0.180
$H^2 = 0.4$						
1	0.825	0.825	0.992	0.992	1.000	1.000
10	0.574	0.671	0.953	0.990	0.997	1.000
50	0.406	0.480	0.890	0.975	0.989	1.000
100	0.340	0.396	0.853	0.955	0.983	0.999
500	0.220	0.248	0.749	0.862	0.959	0.998
1000	0.179	0.198	0.699	0.806	0.944	0.994
